# Simultaneous determination of secondary metabolites from *Vinca rosea* plant extractives by reverse phase high performance liquid chromatography

**DOI:** 10.4103/0973-1296.80662

**Published:** 2011

**Authors:** Mohammad Jamshed Ahmad Siddiqui, Zhari Ismail, Noor Hafizoh Saidan

**Affiliations:** *School of Pharmaceutical Sciences, Universiti Sains Malaysia, P. Penang 11800, Malaysia*

**Keywords:** Catharanthine, isocratic, quality assurance, *Vinca rosea*, vincristine, vindoline, vinblastine

## Abstract

**Background:**

*Vinca rosea* (Apocynaceae) is one of the most important and high value medicinal plants known for its anticancer alkaloids. It is the iota of the isolated secondary metabolites used in chemotherapy to treat diverse cancers. Several high performance liquid chromatography (HPLC) methods have been developed to quantify the active alkaloids in the plant. However, this method may serve the purpose in quantification of *V. rosea* plant extracts in totality.

**Objective:**

To develop and validate the reverse phase (RP)-HPLC method for simultaneous determination of secondary metabolites, namely alkaloids from *V. rosea* plant extracts.

**Materials and Methods:**

The quantitative determination was conducted by RP-HPLC equipped with ultraviolet detector. Optimal separation was achieved by isocratic elution with mobile phase consisting of methanol:acetonitrile:ammonium acetate buffer (25 mM) with 0.1% triethylamine (15:45:40 v/v) on a column (Zorbax Eclipse plus C_18_, 250 mm % 4.6 mm; 5 *μ*m). The standard markers (vindoline, vincristine, catharanthine, and vinblastine) were identified by retention time and co-injected with reference standard and quantified by external standard method at 297 nm.

**Results:**

The precision of the method was confirmed by the relative standard deviation (R.S.D.), which was lower than 2.68%. The recoveries were in the range of 98.09%-108%. The limits of detection (LOD) for each marker alkaloids were lower than 0.20 *μ*g. Different parts of the *V. rosea* extracts shows different concentrations of markers, flower samples were high in vinblastine content, while methanol extract from the leaves contains all the four alkaloids in good yield, and there is no significant presence of markers in water extracts.

**Conclusion:**

HPLC method established is appropriate for the standardization and quality assurance of *V. rosea* plant extracts.

## INTRODUCTION

*Vinca rosea* (L.) G. Don (Apocynaceae) is a medicinal plant better known as Madagascar periwinkle and in Malaysia as Kemuning Cina. The aerial part of the plant contains about 130 different alkaloids from which well-known high value secondary metabolites vincristine and vinblastine are used in chemotherapy to treat diverse cancers, while ajmalicine and serpentine are prescribed for hypertension.[1] A large body of literature documented the activities of *V. rosea* in different ailments.[2–10] Recently, antioxidant potential was assessed against 2,2-diphenyl-1-picrylhydrazyl (DPPH) along with screening of phenolic compounds.[10,11] Since more than three decades, different analytical techniques have been used for qualitative or quantitative determination of *V. rosea* metabolites. Among them high performance liquid chromatography (HPLC) technique is still widely used for the separation and analysis of secondary metabolites from *V. rosea*. Separation of alkaloids by HPLC analysis is not only essential for plant cell line screening, but also for the design and the validation of product recovery and purification processes at an industrial scale. Therefore, efficiency of the harvesting procedure as well as the accuracy of separation methods relies on the detectors sensitivity highlighted the studies on *V. rosea* alkaloids by HPLC.[10,12] The major constraint for this type of studies is the lack of sensitive and accurate rapid estimation methods due to complexity in the chemical assay of molecules that occur in low quantities. HPLC system equipped with an auto sampler provides a powerful tool to analyze various samples. The separation of indole alkaloids is based on reverse phase chromatography using C_18_ column as a stationary phase.[10,13–21] Several mobile phases usually consist of a mixture of buffer solutions like diammonium phosphate[10,22] or ammonium acetate supplemented with triethylamine[10,23] along with methanol or acetonitrile. Detection was carried out using a UV detector at fixed wavelength[10,16] or a fluorescence detector.[10,14] Recently in one of the study Pereira and associates discuss the metabolite analysis and its biological potential using HPLC analysis for phenolic compounds and amino acids of *V. rosea* seeds.[24] The present study is aimed to develop a simple and sensitive method for the simultaneous quantification of alkaloids, which can be used for quality control of herbal products from *V. rosea* and other similar species containing these alkaloids in or around Malaysia region.

## MATERIAL AND METHODS

### Preparation of plant extractives

*V. rosea* plant cultivated and propagated under controlled conditions with the joint venture of USM-UNIMAP at Titi Tinggi, Perlis, Malaysia. Voucher specimens of the plant materials were deposited at Bilik Herba, School of Pharmaceutical Sciences, Universiti Sains Malaysia. The different parts of the *V. rosea* plant (leaves, stem, and flower) were collected and air dried in the month of December 2009 and pulverized into a fine powder using a milling machine (Retsch GmbH, Germany) and extracted with three different types of solvents methanol, methanol:water (1:1), and water, respectively. Soxhlet extractor was used for methanol and methanol:water extractives for 12 h while for water extractives powder was suspended in water bath at 60°C for 6 h. Each extract was concentrated on a rotary evaporator under vacuum and freeze dried. The lyophilized extracts were then kept in freezer prior to use.

### Chemical reagents and materials

The standard markers vincristine and vinblastine were purchased (Calbiochem, EMD Biosciences Inc., CA), whereas catharanthine and vindoline were generously provided by Mr Milind Hanovar (Charms Chem Pvt. Ltd, Pune, India). Ammonium acetate and triethylamine (TEA) and all of the other solvents either of analytical grade or of HPLC grade were purchased from Merck (Darmstadt, Germany). Deionized water for HPLC was prepared using ultrapure water purifier system (Elgastat, Bucks, UK).

### Instrumentation and chromatographic conditions

The high HPLC was performed using an Agilent Technologies Series 1100, Waldronn, Germany) system equipped with degasser (G 1379 A), quaternary pump (G 1311 A), auto sampler (G 1313 A), column oven (G 1316A), and ultraviolet (UV) detector (G 1314 A). The detector was operated at ultraviolet wavelength detection at 297 nm and the sensitivity of the detector was set at 0.005 AUFS. An Agilent Eclipse plus C_18_ (Agilent Technologies, USA) column (5 μm, 250 mm % 4.6 mm, i.d.), fitted with analytical guard column (4.6 × 12.5 mm × 5μm) (Agilent Technologies, USA) was used for the chromatographic separation. The temperature of the column was maintained at 35°C. The injection volume of 10 μL was used. The isocratic mobile phase comprised methanol (solvent A), acetonitrile (solvent B), and 25 mM ammonium acetate with 0.1% triethylamine (solvent C) (15:45:40). Analysis was performed at a flow rate of 1 mL/min and the samples were quantified using peak area for the four alkaloids. Data acquisition was performed by Chemstation software A.08.03 (Agilent Technologies, USA). Standard calibration curves were established by plotting the areas of peaks against different concentrations.

### Standard solutions

Individual stock solutions of vindoline, vincristine, catharanthine, and vinblastine were prepared at a concentration of 5 mg/mL in methanol. These stock solutions were stored at –20°C. The different amounts of concentrations of these stock solutions were used for the preparation of calibration curve, linear in the range of 0.5-200 *μ*g/mL, the regression equations are given in Table 1

**Table 1:** Results of calibration, limits of detection and LOQ of alkaloids Vindoline, Vincristine, Catharanthine and Vinblastine by high performance liquid chromatography

Standards (RT±SD)	Linear regression equation	R^2^	Linear range (μg mL^-1^)	LOD (μg mL^-1^)	LOQ (μg mL^-1^)
Vindoline	y = 7.1445× - 0.0412	1	0.5-200	0.20	1.8
(10.28±0.01)					
Vincristine	y = 11.776× + 0.5164	1	0.5-100	0.25	1.9
(12.46±0.02)					
Catharanthine	y = 7.8493× + 0.5446	1	0.5-200	0.25	2.1
(13.96±0.01)					
Vinblastine	y = 8.9781× -0.9097	0.999	0.5-100	0.20	2.0
(19.59±0.06)					

### Sample preparations

Accurately weighed samples of *V. rosea* extracts (100 mg) were dissolved in 25 mL mixture of (methanol and 1% triethylamine) sonicate (JAC 1002, ultrasonic, KODO Technical Research Co. Ltd, Korea) for 15 min. Make it in a volumetric flask to a known volume. The working samples were filtered through a PTFE 0.45 μm filter (Whatman, Maidstone, England) into an amber glass HPLC vial prior to analysis.

### Statistical analysis

The samples were analyzed in triplicates for and results were averaged. Within day and between days, the accuracy samples were analysed six times and the results were averaged. The extracts from different parts of the plants were analyzed in triplicates and their results were presented as mean ± standard deviation (SD).

### High performance liquid chromatography method validation

The precision and accuracy of the method was performed through within day and between days run validations. Each standard curve was separately constructed on each day of analysis. The within day precision and accuracy were determined for each standard on three concentration with five replicates on a single day. The resulting retention time and peak area were used to calculate the standard deviation and relative standard deviation (RSD %). The accuracy of the method was verified through recovery studies by spiking the standard solution at three different concentration levels. The accuracy was calculated with the value of detection versus added amounts.

The limit of detection was set where the ratio of the standard’s peak area to noise was greater than three. The limit of quantification was determined as the lowest quantifiable concentration with satisfactory between-days and within-day precision and accuracy of less than 20% for both coefficient of variations and percentage error.

Identification of alkaloids from the crude plant extracts were established by comparison with retention time with those of authentic standards. The external standard method was used for the HPLC quantification. The results are reported as mg/g in the crude extract.

## RESULTS AND DISCUSSIONS

**Figure 1:**
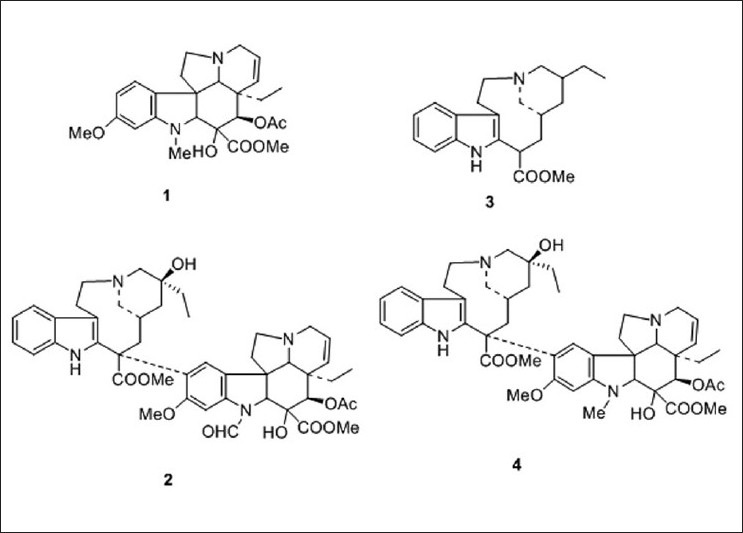
Chemical structure of the marker alkaloids

**Figure 2:**
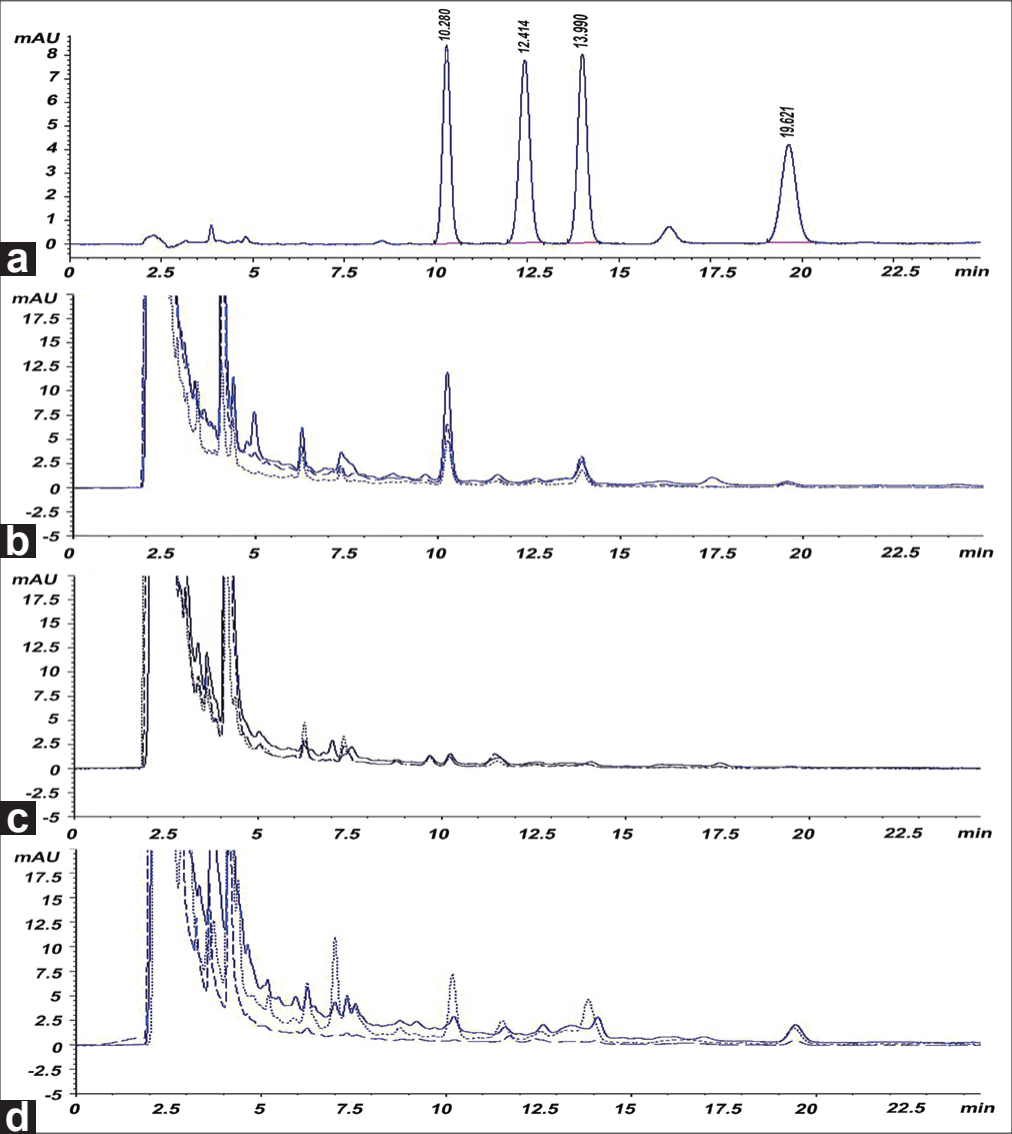
(a) HPLC chromatogram of mixed alkaloid markers; (b) leaves extracts; (c) stem extracts; and (d) flower extracts

All of the structures of standard markers were shown in Figure 1. As all the four markers have good absorption at 297 nm compared with previous methods,[19,21,25] this wavelength was used for quantity. A typical HPLC chromatogram of four standard alkaloids and a product is presented in Figure 2, which showed all the four compounds were eluted within 30 min with satisfactory resolution. The mean retention times [Table 1] for vindoline (1), vincristine (2), catharanthine (3), and vinblastine (4) were 10.28 ± 0.01, 12.46 ± 0.02, 13.96 ± 0.01, and 19.59 ± 0.06, respectively.

The precision under conditions of repeatability was determined by performing six injections of product extract on the same day or 12 injections of the same solution in three different days, respectively. The R.S.D. of intraday was ≤2.68% and R.S.D. of interday was ≤2.21%, indicating repeatability is acceptable. The data were shown in Table 2. Calibration curves were constructed by plotting analyte corrected concentrations with purity against peak areas. A good linearity was achieved in the range 0.5- 200 μg/mL for compound 1 with the determination coefficient (R^2^) = 1; 100 - 0.5 μg/mL for compound 2 with R^2^ = 1; 200 - 0.5 μg/mL for compound 3 with R^2^ = 1; 100 - 0.5 *μ*g/mL for compound 4 with R^2^ = 0.9999. The LODs for all standard alkaloids were ≤ 0.20 *μ*g/mLbetter than previously reported.[25] Data were shown in Table 1

**Table 2:** Recovery, within day and between day precision and accuracy values of vindoline (1), vincristine (2), catharanthine (3) and vinblastine (4)

Conc. ug/ml	Recovery (n=3)		Within day (n=6)		Between day (n=6)	
	Mean%	RSD%	Accuracy%	Precision RSD %	Accuracy%	Precision RSD %
Vindoline (1)						
200	100.06	0.24	99.97	0.15	100.12	0.26
100	99.72	0.3	100.06	0.12	98.3	0.4
20	99.98	0.02	100.04	0.59	98.1	0.67
10	100.68	0.24	98.91	1.14	101.1	0.95
2	108	6.9	103.5	2.68	106.6	2.21
Vincristine (2)					
100	100.05	0.14	100.17	0.05	99.23	0.12
100	100.05	0.14	100.17	0.05	99.23	0.12
10	100.12	0.35	100.07	0.2	101.2	0.25
5	99.56	0.43	99.92	0.06	97.2	0.34
2	99.26	0.15	98.43	1.9	100.1	1.4
1	102.9	0.63	96.33	0.59	103.4	1.12
Catharanthine (3)						
200	99.97	0.06	99.73	0.04	99.98	0.03
100	100.38	0.27	99.55	0.02	101.2	0.23
20	99.75	1.1	99.3	0	97.3	0.56
10	98.09	0.53	98.13	0.05	99.23	0.67
2	107	0.46	110	0.25	105.2	0.12
Vinblastine (4)						
100	100.09	0.17	100.07	0.63	101.1	0.21
10	98.62	1.58	99.33	0.28	97.12	0.32
5	100.35	1.65	100.09	0.34		
2	99.36	0.41	101.26	1.09	99.12	0.12
1	102.36	2.8	105.11	0.19	103.2	1.1

Different crude extracts were analyzed using wavelength 297 nm and the contents were calculated. Results are shown in Table 3. VRPLM contains all the alkaloids in good amount. The most abundant alkaloids vindoline and catharanthine were found in VRPLM, while vinblastine was found to be significantly high in flowers. The results of this study show that methanol is a good solvent for extraction to prepare alkaloids-enriched extracts.

**Table 3:** Contents of alkaloids vindoline (1), vincristine (2), catharanthine (3) and vinblastine (4) indifferent parts of *V. rosea* extracts

Sample	Extraction	Contents of alkaloids (mg g-1 ± SD)			
	Solvent	1	2	3	4
VRPLM	MeOH	5.1 ± 0.5	0.13 ± 0.1	1.5 ± 0.2	0.18 ± 0.1
VRPLMW	MeOH: Water (1:1)	2.4 ± 0.12	0.15 ± 0.2	1.7 ± 0.01	0.2 ± 0.01
VRPLW	Water	1.3 ± 0.01	0.13 ± 0.3	0.5 ± 0.001	0.2 ± 0.1
VRPSM	MeOH	0.4 ± 0.6	nd	nd	nd
VRPSMW	MeOH: Water (1:1)	0.3 ± 0.1	0.1 ± 0.002	0.2 ± 0.01	0.1 ± 0.005
VRPSW	Water	0.3 ± 0.01	0.08 ± 0.1	0.1 ± 0.1	0.2 ± 0.01
VRPFM	MeOH	3.1 ± 0.2	0.2 ± 0.01	0.7 ± 0.6	1.2 ± 0.5
VRPFMW	MeOH : Water (1:1)	0.7 ± 0.01	0.2 ± 0.03	0.3 ± 0.4	0.3 ± 0.01
VRPFW	Water	0.7 ± 0.001	nd	nd	0.2 ± 0.1

## CONCLUSION

The screening of secondary metabolites from *V. rosea* is always a difficult task but compared with previous studies this method seems to be more reliable and versatile in the qualitative and quantitative evaluation of *V. rosea* plant extractives. For the sample preparation using 1% triethylamine in methanol as extraction solvent may be helpful in rapid preparation of crude extracts compared with previous tedious extraction methods.[21] Hence, this method can be applied for the standardization and quality assurance or pharmacokinetics studies of *V. rosea* extracts.

## References

[1] Padua LS, Bunyapraphatsara N, Lemmens RH (1999). Plant resources of South-East Asia No.12. Medicinal and poisonous plants 1:Prosea Foundation, Bogor, Indonesia.

[2] Taylor WI, Fransworth NR (1975).

[3] Chattopadhyay RR (1999). A comparative evaluation of some blood sugar lowering agents of plant origin. J Ethnopharmacol.

[4] El-Sayed A, Cordell GA (1981). Catharanthus alkaloids. XXXIV. Catharanthamine, a new antitumor bisindole alkaloid from Catharanthus roseus. J Nat Prod.

[5] El-Sayed A, Handy GA, Cordell GA (1983). Catharanthus alkaloids, XXXVIII.Confirming structural evidence and antineoplastic activity of the bisindole alkaloids leurosine-N’b-oxide (pleurosine), roseadine and vindolicine from Catharanthus *roseus*. J Nat Prod.

[6] Johnson IS, Armstrong JG, Gorman M, Burnett JP (1963). The Vinca alkaloids: A new class of oncolytic agents. Cancer Res.

[7] Ueda JY, Tezuka Y, Banskota AH, Le Tran Q, Tran QK, Harimaya Y (2002). Antiproliferative activity of vietnamese medicinal plants. Biol Pharma Bull.

[8] Singh SN, Vats P, Suri S, Shyam R, Kumria MM, Ranganathan S (2001). Effect of an antidiabetic extract of *Catharanthus roseus* on enzymic activities in streptozotocin induced diabetic rats. J Ethnopharmacol.

[9] Heijden RV, Jacobs DI, Snoeijer W, Hallard D, Verpoorte R (2004). The Catharanthus alkaloids: Pharmacognosy and biotechnology. Curr Med Chem.

[10] Zhou ML, Shao JR, Tang YX (2009). Production and metabolic engineering of terpenoid indole alkaloids in cell cultures of the medicinal plant *Catharanthus roseus* (L.) G. Don (Madagascar periwinkle). Biotechnol Appl Biochem.

[11] Ferreres F, Pereira DM, Valentao P, Andrade PB, Seabra RM, Sottomayor M (2008). New phenolic compounds and antioxidant potential of *Catharanthus roseus*. J Agric Food Chem.

[12] Hisiger S, Jolicoeur M (2007). Analysis of *Catharanthus roseus* alkaloids by HPLC. Phytochem Rev.

[13] Tikhomiroff C, Allais S, Klvana M, Hisiger S, Jolicoeur M (2002). Continuous selective extraction of secondary metabolites from *Catharanthus roseus* hairy roots with silicon oil in a two-liquid-phase bioreactor. Biotechnol Prog.

[14] Renaudin JP (1984). Reversed-phase high-performance liquid chromatographic characteristics of indole alkaloids from cell suspension cultures of *Catharanthus roseus*. J Chromatogr A.

[15] Bhadra R, Vani S, Shanks JV (1993). Production of indole alkaloids by selected hairy root lines of *Catharanthus roseus*. Biotechnol Bioeng.

[16] Barthe L, Ribet JP, Pélissou M, Degude MJ, Fahy J, Duflos A (2002). Optimization of the separation of Vinca alkaloids by nonaqueous capillary electrophoresis. J Chromatogr A.

[17] Naaranlahti T, Nordström M, Huhtikangas A, Lounasmaa M (1987). Determination of Catharanthus alkaloids by reversed-phase high-performance liquid chromatography. J Chromatogr A.

[18] Zhou H, Tai Y, Sun C, Pan Y (2005). Rapid identification of vinca alkaloids by direct-injection electrospray ionisation tandem mass spectrometry and confirmation by high-performance liquid chromatography-mass spectrometry. Phytochem Anal.

[19] Zhao J, Zhu W, Hu Q (2001). Enhanced catharanthine production in *Catharanthus roseus* cell cultures by combined elicitor treatment in shake flasks and bioreactors. Enzyme Microb Technol.

[20] Uniyal GC, Bala S, Mathur AK, Kulkarni RN (2001). Symmetry C18 column: A better choice for the analysis of indole alkaloids of *Catharanthus roseus*. Phytochem Anal.

[21] Singh DV, Maithy A, Verma RK, Gupta MM, Kumar S (2000). Simultaneous determination of catharanthus alkaloids using reversed phase high performance liquid chromatography. J Liquid Chromatogr Relat Technol.

[22] Renaudin JP (1985). Extraction and fluorimetric detection after high-performance liquid chromatography of indole alkaloids from cultured cells of *Catharanthus roseus*. Phsiol Veg.

[23] Dagnino D, Schripsema J, Verpoorte R (1996). Analysis of several iridoid and indole precursors of terpenoid indole alkaloids with a single HPLC run. Planta Med.

[24] Pereira DM, Ferreres F, Oliveira J, Valentão P, Andrade PB, Sottomayor M (2009). Targeted metabolite analysis of *Catharanthus roseus* and its biological potential. Food Chem Toxicol.

[25] Tikhomiroff C, Jolicoeur M (2002). Screening of *Catharanthus roseus* secondary metabolites by high-performance liquid chromatography. J Chromatogr A.

